# Melanogenesis Promoting Effect, Antioxidant Activity, and UPLC-ESI-HRMS Characterization of Phenolic Compounds of Argan Leaves Extract

**DOI:** 10.3390/molecules26020371

**Published:** 2021-01-12

**Authors:** Thouria Bourhim, Myra O. Villareal, François Couderc, Abdellatif Hafidi, Hiroko Isoda, Chemseddoha Gadhi

**Affiliations:** 1 Faculty of Sciences Semlalia, Cadi Ayyad University, Avenue Prince Moulay Abdellah, BP 2390, 40000 Marrakesh, Morocco; t.bourhim@gmail.com (T.B.); a.hafidi@uca.ac.ma (A.H.); 2 Alliance for Research on the Mediterranean and North Africa (ARENA), University of Tsukuba, Tennodai 1-1-1, Tsukuba City 305-8572, Japan; villareal.myra.o.gn@u.tsukuba.ac.jp; 3 Faculty of Life and Environmental Sciences, University of Tsukuba, Tennodai 1-1-1, Tsukuba City 305-8572, Japan; 4 UMR CNRS 5623 (National Center for Scientific Research), Paul Sabatier University, 118 Route de Narbonne, F-31062 Toulouse, France; couderc@chimie.ups-tlse.fr

**Keywords:** argan leaves, melanogenesis stimulation, antioxidant activity, phenolic compounds, UPLC-ESI-HRMS

## Abstract

The use of natural products for the regulation of skin pigmentation is gaining popularity. In the present study, we evaluated the effect of argan leaves extract (ALE) on melanogenesis in B16 melanoma cells, determined its antioxidant activity, then quantified and identified its phenolic components. B16 cells were treated with various concentrations of ALE, then the cell viability and proliferation were assessed using MTT assay while the melanin content was determined using spectrophotometric methods. The expression level of tyrosinase (TYR), tyrosinase related protein-1 (TRP-1) and dopachrome tautomerase (DCT) was evaluated by Western blotting. The antioxidant activity of ALE was investigated using four different assays while UPLC-ESI-HRMS analysis was used to characterize the ALE phenolic profile. Fourteen phenolic compounds were identified, of which six are reported for the first time to be present in ALE. ALE treatment increases the melanin content of B16 cells in a dose-dependent manner without cytotoxicity. This was revealed by the observed ALE-increased expression level of TYR, DCT, and TRP-1. These bioactivities may be mainly attributed to its high flavonoids content. Argan leaves have the potential for use as a treatment for hypopigmentation disorders and as a bioactive component of cosmetic products that aim to increase pigmentation.

## 1. Introduction

Melanin is a pigment produced by melanocytes that provides the skin with protection against harmful damage caused by ultraviolet (UV) sunlight, toxic drugs, and chemicals [1,2]. In addition, this pigment gives the skin its characteristic color such that any alteration in skin pigmentation may impact the individual’s appearance, and cause significant emotional and psychological distress, as well as reduced quality of life [3]. Thus, the increasing demand for treatments to regulate skin pigmentation is a great clinical challenge for both physicians and scientists.

Among skin disorders, skin hypopigmentation, such as post-inflammatory hypopigmentation, pityriasis alba, or progressive macular hypomelanosis, is an embarrassing dyschromia that is particularly visible in dark-skinned individuals [4], and which still needs effective treatments [3]. These disorders have been linked to deficiency in melanin synthesis [5], an alteration in melanosomes maturation and distribution [6], or a defect in melanocytes function leading to a decrease in melanocytes viability and/or activity [7].

The promotion of melanin synthesis in the skin using natural products as a treatment for hypopigmentation disorders has gained attention in the cosmetics field. Melanogenesis is a multi-step biosynthetic pathway that utilizes l-tyrosine to produce brown/black melanin (eumelanin) and red/yellow melanin (pheomelanin) through the catalytic activities of melanogenesis enzymes tyrosinase (TYR), tyrosinase-related protein 1 (TRP1), and dopachrome tautomerase (DCT). TYR catalyzes the conversion of l-tyrosine into l-DOPA, the rate-limiting step in melanin synthesis [7], and then l-DOPA to dopaquinone. The conjugation of dopaquinone with cysteine or glutathione produces pheomelanin through a series of reactions. In the absence of thiol compounds, dopaquinone may spontaneously oxidize to dopachrome which is transformed by DCT into 5,6-dihydroxyindole-2-carboxylic acid (DHICA). TRP1 catalyzes the oxidation of DHICA to indole-5,6-quinone-2-carboxylic acid, which will then polymerize to form eumelanins [8].

*Argania spinosa* (Sapotaceae) is a tree endemic to Morocco, and known worldwide for its oil, for its cosmetic properties and various skin applications. Traditionally, beside the oil, other parts of the argan tree including the leaves, roots, press-cake, kernels, and fruit pulp have been used for centuries by the local population [9]. Argan leaves are specifically valued for their tanning and medicinal properties [9].

Previously, we have reported that argan oil and argan press-cake have an anti-melanogenesis effect on B16 melanoma cells by decreasing *Mitf* and the expression of melanogenic enzymes [10,11]. The present study determined the effect of argan leaves extract (ALE) on melanogenesis and evaluated its antioxidant activities, quantified the phenolic compounds present in ALE, and characterized these compounds by UPLC-ESI-HRMS analysis.

## 2. Results

### 2.1. ALE Had No Cytotoxic Effect on B16 Melanoma Cells

**Figure 1:**
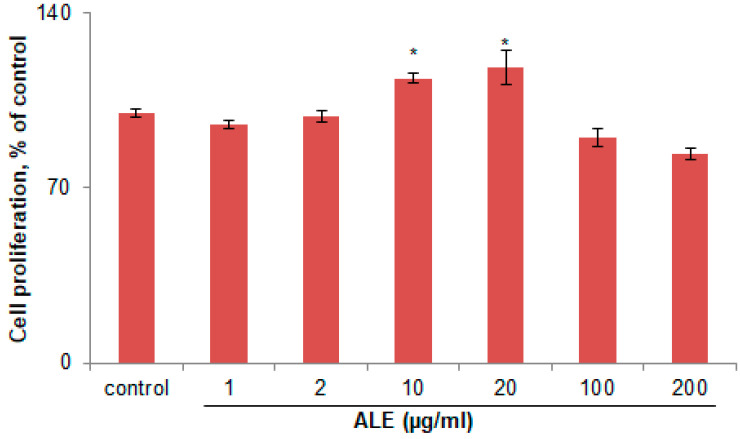
Cytotoxicity of argan leaves extract (ALE) on B16 cells. Cells were incubated with ALE at different concentrations (1, 2, 10, 20, 100, and 200 µg/mL) for 48 h. Values represent the percentage of cell proliferation relative to the control, expressed as mean ± standard deviation (SD) of three independent experiments. * Statistically significant (*p* ≤ 0.05) versus the non-treated control (ANOVA, Tukey’s test).

The cytotoxicity of ALE on B16 melanoma cells was evaluated by performing 3-(4,5-dimethylthiazol-2-yl)-2,5-diphenyl-tetrazolium bromide (MTT) assay (Figure 1). Treatment with ALE at different concentrations (1–200 µg/mL) for 48 h had no cytotoxic effect on B16 melanoma cells.

### 2.2. ALE Stimulated Melanogenesis in B16 Cells

**Figure 2:**
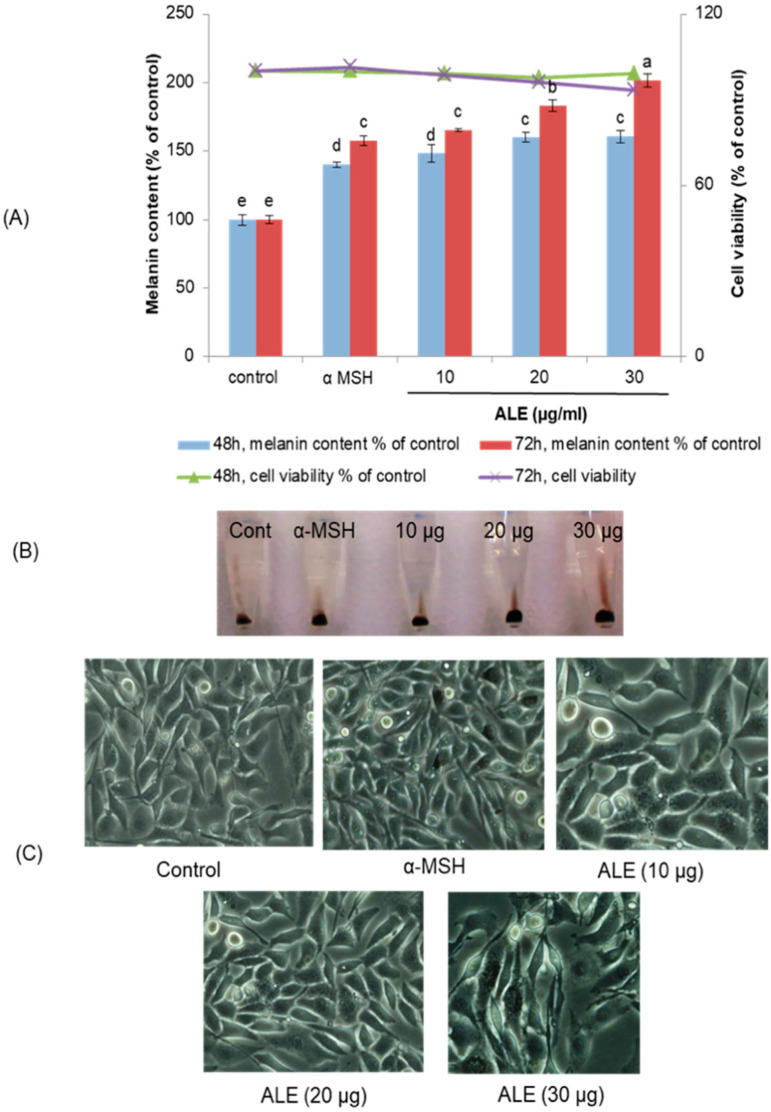
Melanin content and cell viability of argan leaves extract (ALE)-treated or α MSH-treated B16 cells. Cells were treated without (control) or with α-MSH (0.2 µM) or ALE (10, 20 and 30 µg/mL) for 48 h and 72 h. (**A**) Melanin content of the cells treated with ALE. Cells treated with 0.2 µM α-MSH served as a positive control. The synthesized melanin and total number of cells were measured as described in Materials and Methods. The bar graph indicates the melanin content (left-hand y-axis) while the line graph indicates cell viability (right-hand y-axis). The results are presented as the mean ± SD of three independent experiments; different letters on the histograms indicate significant difference between treatments (*p* ≤ 0.05) (ANOVA Tukey’s test). (**B**) Photograph of extracted melanin pellet from B16 cells treated without (control) or with α-MSH (0.2 µM), or ALE (10, 20 and 30 µg/mL). (**C**) Images of B16 cells treated without or with ALE or α-MSH (400× magnification).

The melanin content of ALE-treated cells was significantly increased in a time- and dose-dependent manner without cytotoxicity, compared to the control group (Figure 2A). Melanin content in B16 cells treated for 72 h with 10, 20, and 30 µg/mL of ALE was increased by 65%, 83%, and 101%, respectively. When compared to the positive control, α-melanocyte-stimulating hormone (α-MSH), which increased the melanin content in melanoma cells by 58% vs. control, ALE at 20 and 30 µg/mL had higher melanogenesis stimulation activity. The color of the melanin pellet from ALE-treated B16 cells clearly showed that the melanin content was markedly increased by ALE (Figure 2B). Moreover, ALE did not cause any changes to the morphology of B16 cells (Figure 2C).

### 2.3. ALE Modulated the Melanogenic Enzymes Expression in B16 Cells

**Figure 3:**
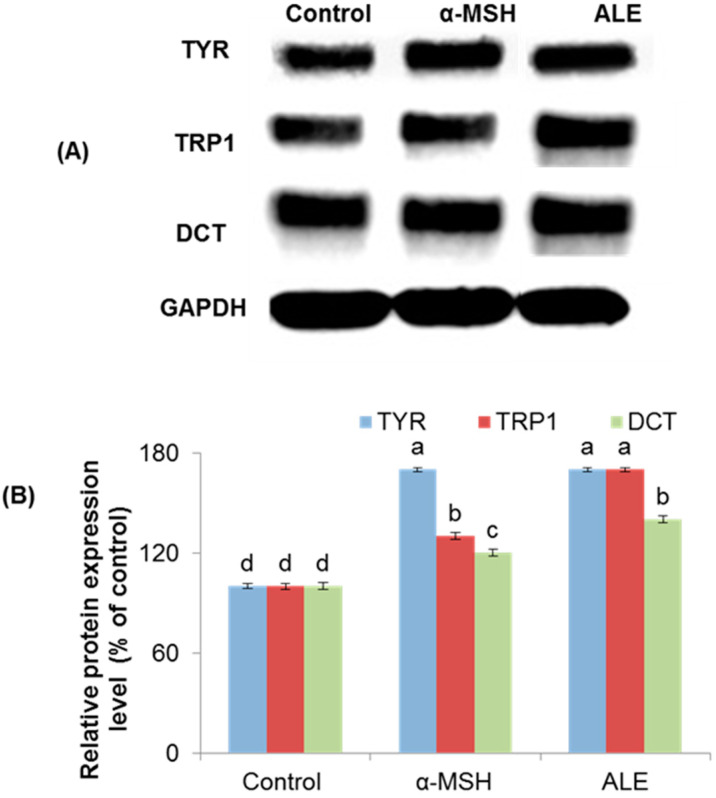
Effect of argan leaves extract (ALE) on melanogenic enzymes TYR, TRP1, and DCT expression level, determined by Western blot analysis. Western blot bands (**A**) and the corresponding densitometric values (**B**) are presented. B16 melanoma cells were treated without (Control) or withα-MSH (0.2 µM) or ALE (30 µg/mL) and incubated for 72 h. Values are mean ± SD derived from three independent experiments; different letters on the histograms indicate significant difference between treatments (*p* ≤ 0.05) (ANOVA Tukey’s test). The protein bands intensities of TYR, TRP1, and DCT were obtained using Li-COR Software.

The protein expression level of the three melanogenic enzymes, TYR, TRP1, and DCT in ALE-treated cells was determined via Western blot analysis. ALE markedly increased the expression level of those three enzymes in a time-dependent manner (Figure 3A). The analysis of densitometric values of proteins expressions showed that TYR, TRP1, and DCT expression level was significantly increased by 80%, 60%, and 20%, respectively, after 72 h (Figure 3B). The positive control α-MSH, as expected, significantly increased the expression level of TYR, TRP1, and DCT by 70%, 30%, and 20%, respectively, for the same treatment time period.

### 2.4. Antioxidant Activity of ALE

The results of different antioxidant assays showed that ALE exhibited important 1,1-diphenyl-2-picrylhydrazyl (DPPH) and 2,2’-azinobis-3-ethylbenzothiazoline-6-sulphonate (ABTS) free radicals scavenging activities (IC_50_ = 0.508 ± 0.016 mg/mL, and IC_50_ = 0.373 ± 0.001 mg/mL, respectively) (Table 1). This antioxidant activity of ALE, however, is still lower than that of butylated hydroxytoluene (BHT) or ascorbic acid. The ability of ALE to reduce Fe^3+^/ferricyanide complex to the ferrous form was also evaluated. The reducing power of ALE (IC_50_ = 0.316 ± 0.001 mg/mL) was higher than that of BHT (IC_50_ = 0.354 ± 0.011 mg/mL), while ascorbic acid showed higher activity (IC_50_ = 0.070 ± 0.011 mg/mL). The β-carotene bleaching assay was used to estimate the inhibition of linoleic acid’s peroxidation by ALE and the results showed that ALE exhibited significant lipoperoxidation inhibitory activity (IC_50_ = 1.679 ± 0.070 mg/mL), which was lower than that of BHT.

### 2.5. Quantification and Characterization of ALE Phenolic Compounds

**Figure 4:**
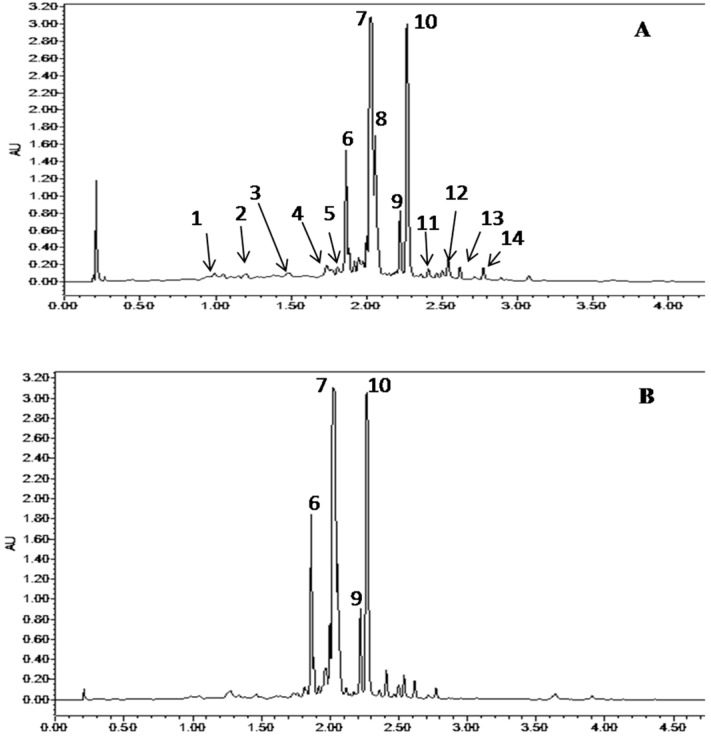
UV chromatograms of argan leaves analyzed by UPLC-ESI-HRMS (**A**) at 280 nm and (**B**) 320 nm. The different compounds were identified during the same run with the ESI-HRMS spectra. 1: Gallic acid; 2: (+)-Gallocatechin; 3: (−)-Epigallocatechin; 4: (+)-Catechin; 5: (−)-Epicatechin; 6: Quercetin-3-*O*-Glucuronide; 7: Myricetin-3-*O*-Galactoside; 8: Unknown; 9: Myricitrin; 10: Quercitrin; 11: Quercetin-7-*O*-rhamnoside; 12: Myricetin; 13: Rutin (Quercetin-3-*O*-rutinoside); 14: Quercetin.

The spectrophotometric quantification of phenol content (Table 1) showed that ALE has a high total phenol content (TPC) of 198.61 ± 0.20 tyrosol equivalent (TYE) mg/g dry matter (DM)) and a significant total flavonoid content (TFC) (128.41 ± 1.58 catechin equivalent (CAE) mg/g DM). Among the flavonoid subgroups, flavanols and proanthocyanidins contents were 59.59 ± 1.55 CAE mg/g DM and 6.85 ± 0.13 cyanidin equivalent (CYE) mg/g DM, respectively. The phenolic compounds that were identified using UPLC-ESI-HRMS analysis are summarized in Table 2. Component identification was based on the analysis of their retention times, exact mass spectra, and comparison with the data reported in the literature. A total of 14 polyphenols including catechins, flavonoids, and phenolic acids (Figure 4) were identified in ALE. The major phenolic compounds identified in ALE were quercetin-3-*O*-glucuronide, myricetin-3-*O*-galactoside, myricitrin, and quercitrin.

## 3. Discussion

Skin pigmentation represents one of the most notable physical traits in humans, thus any alteration, as in the case of hypopigmentation can cause significant emotional and psychological distress and reduced quality of life [3]. Hypopigmentation disorders are usually treated with topical creams or oral medication to restore skin color but most of these treatments have unwanted side effects [20]. Thus, the discovery of a safe product that can promote melanin production and also possesses antioxidant properties is of a great interest. Within this context, the antioxidant activities of ALE and its melanogenesis effect on B16 melanoma cells were evaluated.

The results of the MTT assay showed that ALE did not significantly affect cell viability at all tested ALE concentrations, indicating that it is non-cytotoxic to B16 cells. The quantification of melanin content revealed that ALE significantly enhances melanin synthesis in a concentration- and time-dependent manner. Moreover, ALE appears to be more effective than α-MSH in stimulating melanogenesis (*p* < 0.001). ALE at a concentration of 30 µg/mL increased the cells melanin content by almost two-fold compared to α-MSH-treated cells. Melanin biosynthesis is catalyzed by three melanocytes-specific enzymes, TYR, TRP1, and DCT [1]. The expression of these enzymes was significantly increased by ALE compared to the control. Therefore, ALE’s melanogenesis-stimulating effect was attributed to the elevated expression of these three key enzymes.

To evaluate the antioxidant activity of an extract, one test is not sufficient since various mechanisms are involved in the antioxidant action such as scavenging of free radicals, sequestering transition metal ions, inhibition of lipid peroxidation, etc. [21]. Therefore, in this study, four different tests representing different antioxidant mechanisms were used: DPPH and ABTS free radical scavenging assays in which the ability of ALE to reduce both radicals by transferring, respectively, a hydrogen or an electron was assessed; the β-carotene/linoleic acid bleaching assay, in which the potential of ALE to delay lipid peroxidation was evaluated; and the reducing power assay, in which the potential of ALE to reduce iron ion Fe^3+^ to Fe^2+^ was appraised.

The results showed that ALE may be a promising and significant source of natural antioxidants. The high antioxidant activity of ALE is most likely due to its high total phenol content (198.61 mg TYE/g DM), especially its total flavonoids content (128.41 mg CAE/g DM) represented by flavanols (59.59 mg CAE/g DM) and proanthocyanins (6.85 mg CYE/g DM). The obtained values are within the range of values reported elsewhere [22,23,24,25,26,27,28]. The total polyphenol content of argan leaves was reported to vary from 29.9 to 240 mg gallic acid equivalent (GAE)/g DM [22,23,24,25,26,27,28].

There are some limitations that should be considered when comparing results of these studies with regard to the content and composition of polyphenols in argan leaves and their antioxidant activity. The synthesis of the secondary metabolites by plants is an ingenious strategy used by plants to control their environment and to adapt to biotic and abiotic conditions [29]. Therefore, the geographic origin, the period of the collection of plant material, and argan tree phenotype are major parameters that impact the chemical composition of the plant [23,25,27,30,31].

The other limitation is that polyphenols extraction and quantification can vary significantly according to the solvent used, the method of extraction (Soxhlet, sonication, maceration), duration of the extraction, and experimental protocol [30,32,33].

The effect of these variants on polyphenol content and composition explains the discrepancy observed among reported values and therefore, comparing them becomes irrelevant. Despite this, all the reports combined explain the richness of polyphenols in argan leaves compared to other medicinal plants [27], which give them their antioxidant properties.

Fourteen phenolic compounds were identified in ALE, of which six compounds have not yet been reported to be present in ALE: gallic acid, (+)-gallocatechin, (−)-epigallocatechin, quercetin-3-*O*-glucuronide, quercetin-7-*O*-rhamnoside, and quercetin. Myricetin-3-*O*-galactoside, myricitrin, quercitrin, myricetin, (+)-catechin, (−)-epicatechin, and rutin (quercetin-3-*O*-rutinoside) have already been reported to be present in argan leaves [16,17,22]. Other phenolic compounds, such as hyperoside have not been detected in the ALE used in this study but their presence in ALE has been reported [16,17,22]. The detection of previously unreported compounds could be due to the differences in the phenotype, and/or geographical origin of the argan leaves [31].

The presence of different phenolic compounds representing different structures in ALE is a possible explanation for not only its high antioxidant activity, but also for its melanogenesis enhancing effect.

Flavonoid chemical structures have been reported to have melanogenesis-stimulating actions [34]. Flavonoids including quercetin, kaempferol, rhamnetin, apigenin, luteolin, chrysin, genestein showed melanogenesis-promoting actions [34,35,36] but rutin, robinetin, myricetin, ipriflavone, epigalocatechin gallate (EGCg) and naringin did not [34].

Quercetin, which has iron-chelating and iron-stabilizing properties [34], has been reported to be a powerful inducer of melanogenesis in human melanocytes [35]. Moreover, some studies have shown that a hydroxyl group bound to the phenyl group of flavonoids plays an important role in stimulating melanogenesis [35,36,37].

The human skin is exposed to UV radiation (UVR) on a daily basis, which results in an increase in the production of reactive oxygen species (ROS) that eventually will cause oxidative stress at the cellular level. ROS cause oxidation of DNA bases that lead to the formation of highly mutagenic lesions such as 8-oxo-deoxyguanine [38]. Melanin provides the first line of defense against UVR by blocking its penetration in the skin [39]. This protective role of increased melanin in the skin is demonstrated by the inverse correlation between skin pigmentation and the incidence of sun-induced skin cancers [40]. Although melanin protects from UVR, it can only absorb 50–75% of the UVR [41]. The use of natural products, such as those present in argan leaves extract, could be useful since aside from promoting melanogenesis, they can also attenuate some of the effect of UVR that the melanin cannot absorb. A lot of bioactive compounds with anti-oxidant properties, such as quercetin, have been reported to regulate melanogenesis, but the direct relationship between the anti-oxidant effect and melanogenesis regulatory effect have not been well-explained yet. The results presented here, however, suggest that the melanogenesis stimulation activities of ALE could be associated with its antioxidant effect. The presence of the flavonoids chemical group, and their synergistic interaction with other ALE compounds, provides ALE with both melanogenesis promotion effect and antioxidant properties making it a promising therapeutic agent against hypopigmentation and UV-induced oxidative stress in the skin.

## 4. Materials and Methods

### 4.1. Chemicals and Reagents

Cell culture and cell-based bioassay reagents Dulbecco’s modified eagle’s medium (DMEM), fetal bovine serum (FBS), 4 mM l-glutamine, 3-(4,5-dimethylthiazol-2-yl)-2,5-diphenyl-tetrazolium bromide (MTT), trizma base, trypsin/ethylene-diamine-tetra-acetic acid (EDTA), α-melanocyte-stimulating hormone (α-MSH), and protease inhibitor cocktail were purchased from Sigma-Aldrich (St. Louis, MO, USA) while penicillin/streptomycin was purchased from Lonza (Alpharetta, GA, USA). Western blot reagents tetramethyl ethylene diamine (TEMED) were purchased from Amersham Bioscience (Uppsala, Sweden); ammonium persulfate (APS) and protein rainbow marker were purchased from GE Healthcare (Buckinghamshire, UK); sodium dodecyl sulfate (SDS) was purchased from GE Healthcare (Danderyd, Sweden); and lysis buffer (RIPA buffer) was obtained from Promega (Madison, WI, USA). For the composition analysis and other cell-free assays, acetonitrile, potassium ferricyanide, 2-thiobarbituric acid, linoleic acid, ferric chloride (FeCl_3_), l-ascorbic acid, Tween 20, β-carotene, 1,1-diphenyl-2-picrylhydrazyl radical (DPPH), 2,2’-azinobis-3-ethylbenzothiazoline-6-sulphonate (ABTS), potassium persulfate, aluminum chloride, potassium hydroxide, ferrous sulphate (FeSO_4_), butylated hydroxytoluene (BHT), tyrosol, catechin, cyanidin, Folin-Ciocalteu reagent, anhydrous sodium carbonate, *p*-(dimethylamino)-cinnamaldehyde (DMACA), trichloroacetic acid, sodium phosphate dibasic and monosodium phosphate were purchased from Sigma-Aldrich (USA); dithiothreitol (DTT) was purchased from Amersham Bioscience (Sweden). Solvents were purchased from Sigma-Aldrich (Munich, Germany). All chemicals and solvents used were of analytical or HPLC grade.

### 4.2. Preparation of ALE

Argan leaves were collected in July 2012 from trees with fusiform fruits in the Sidi Ifni region (southwest of Morocco), and verified by Prof. Ahmed Ouhammou, head of the regional herbarium MARK of the Faculty of Sciences Semlalia, Marrakech, Morocco. Voucher samples (MARK10888-2) are kept in the herbarium of the same institution. They were dried in the dark at 25 °C. Ten grams (10 g) of argan leaves powder was extracted with 100 mL of 70% ethanol for 2 weeks at room temperature. The obtained extract was then centrifuged (1000× *g*, 15 min) and the supernatant was filter-sterilized using a 0.22 µm pore size filter (Millipore, Burlington, MA, USA), and stored at −80 °C until use.

### 4.3. Cell Culture

B16 murine melanoma cells were obtained from the Riken Cell Bank (Tsukuba, Japan) and maintained as a monolayer culture in DMEM supplemented with 10% FBS, 4 mM l-glutamine, 50 units⁄mL penicillin, and 50 µg/mL streptomycin, and incubated at 37 °C in a humidified incubator with 5% CO_2_. Photographs of the cells were taken using a Leica DFC290 HD camera (Beckman Coulter, CA, USA).

### 4.4. Cell Viability Assay

Cell viability and proliferation were investigated using MTT assay [10]. In brief, after seeding and incubating the B16 cells in 96-well plates at 3 × 10^3^ cells per well for 24 h, the culture medium was replaced with fresh culture medium containing ALE at various concentrations (1, 2, 10, 20, 100 and 200 µg/mL). After incubation for 48 h, 10 µL MTT solution (5 mg/mL) was added. The plates were then covered with aluminum foil and incubated at 37 °C for an additional 6 h. SDS (10%) was added to dissolve the formazan crystals. After overnight incubation at 37 °C, the absorbance was measured at 570 nm using a microplate reader (Powerscan HT, Dainippon Pharmaceuticals USA Corporation, NJ, USA). Blanks, containing only medium, MTT, and SDS were used to correct the absorbance. The absorbance of the control cells was set as 100% cell proliferation.

### 4.5. Measurement of Melanin Content

The melanin content of B16 cells was investigated using the method described in Villareal et al. [36]. The cells were seeded onto 100-mm dishes at a density of 5 × 10^5^ cells per 100-mm petri dish and incubated overnight for adhesion. The medium was replaced with medium containing α-MSH (0.2 µM) or ALE (0, 10, 20, 30 µg/mL) and incubated for 48 h or 72 h. After removing the medium, the cells were harvested by trypsinization. The harvested cells were then centrifuged and the growth medium discarded. The pelleted cells were lysed by sonication after addition of 0.1% Triton X-100. The melanin was then precipitated by addition of 10% trichloroacetate and dissolved in 8 N NaOH with incubation for 2 h at 80 °C. The absorbance of the supernatant was measured at 410 nm and the melanin content was determined using a standard curve for synthetic melanin. The results were expressed as a percentage of the control. The cell viability and total cell count were measured using the ViaCount Program of Guava PCA (GE Healthcare, UK) following the manufacturer’s instructions.

### 4.6. Western Blot

B16 melanoma cells were seeded at a density of 5 × 10^5^ cells per 100-mm petri dish and cultivated overnight. The medium was then replaced by medium without (control) or with α-MSH (0.2 µM) or ALE (30 µg/mL). After incubation of B16 cells for 48 h or 72 h, RIPA buffer, containing a protease inhibitor cocktail, was used to harvest the total protein. Extracted protein samples (10 µg) were separated via SDS-PAGE, and transferred onto PVDF membranes. These membranes were incubated with anti-TYR, anti-TRP1, anti-DCT, or anti-GAPDH primary antibodies (Santa Cruz Biotechnology, Santa Cruz, CA, USA); then detected by adding anti-mouse or anti-goat secondary antibodies (LI-COR Biosciences, Lincoln, NE, USA). Proteins were visualized using the LiCor Odyssey Infrared Imaging System (LI-COR Biosciences, Lincoln, NE, USA).

### 4.7. Evaluation of ALE Antioxidant Activity

The antioxidant activity of ALE was determined using different, previously described, antioxidant assays such as DPPH radical scavenging activity, ABTS^+^ cation radical scavenging activity, β-carotene/linoleic acid bleaching assay, and reducing power. All tests were carried out in triplicate and the results were expressed as the mean IC_50_ in mg/mL ± SD of triplicates.

#### 4.7.1. DPPH Radical Scavenging Assay

DPPH stable free radical scavenging assay was performed as previously described by Von Gadow et al., with some modifications [42]. Free radical working solution was prepared by dissolving 4 mg of DPPH* in 100 mL of methanol. ALE (100 µL) at different concentrations was mixed with 3 mL of DPPH* working solution. After 60 mn incubation, the absorbance was read at 517 nm. The scavenging activity, corresponding to the inhibition of DPPH discoloration, was determined by the following Equation (1):

% Inhibition = 100 × [(Ac − As)/Ac](1)

where As represents the sample absorbance and Ac is the control absorbance. The experiment was repeated three times. BHT and ascorbic acid were used as standard controls. The IC_50_ value was calculated from the graph plotting inhibition percentage against extract concentration, and denoted the concentration of sample required to scavenge 50% of DPPH radicals.

#### 4.7.2. Scavenging Activity on ABTS^+^ Radical

The ABTS^+^ radical scavenging activity assay was carried out using the method described by Li et al., with slight modifications [43]. ABTS^+^ radical cation was generated by a reaction between 7 mmol/L ABTS and 2.45 mmol/L potassium persulfate. The reaction mixture was allowed to stand in the dark for 16 h at room temperature. The working solution was prepared by diluting the previous solution with ethanol to an absorbance of 0.700 ± 0.020 at 734 nm before use. The diluted sample (50 µL) was mixed with 1.9 mL of the diluted ABTS^+^ solution. The absorbance value was determined at 734 nm after 6 min of the reaction. BHT and ascorbic acid were used as positive controls.

The inhibition percentage of the radical ABTS^+^ was determined according to Equation (1). The IC_50_ values were calculated as the concentrations providing 50% inhibition of initial ABTS^+^ radical.

#### 4.7.3. β-Carotene/Linoleic Acid Bleaching Assay

The β-carotene/linoleic acid bleaching assay was performed following the method described by Miraliakbari and Shahidi, with slight modification [44]. A mixture of β-carotene and linoleic acid was prepared by dissolving 0.5 mg β-carotene in 1 mL chloroform and 25 µL linoleic acid in 200 mg Tween 20. The chloroform was then completely evaporated under vacuum, and 100 mL of distilled water was subsequently added to the residue, and then the mixture was shaken vigorously to form an emulsion. From this emulsion, 2.5 mL was transferred into different test tubes containing 350 µL of ALE at different concentrations. All samples were vortexed for 1 min and placed at 50 °C in a water bath for 2 h together with a negative control (blank), which contained the same volume of ethanol instead of the samples. The absorbance of samples was measured at 470 nm using a spectrophotometer at initial time (t = 0) against a blank (emulsion without β-carotene). A standard BHT was used as a positive control. Antioxidant activities (inhibitions percentage, I%) of the samples were calculated using the following Equation (2):

% Inhibition = [1 − (A _sample t=0_ − A _sample t= 2h_)/(A _control t=0_ − A _control t=2h_)] ×100 (2)

where A _sample t=0_ and A _sample t=2h_ are the absorbance values for the test sample at the beginning of the experiments and after 2 h assay, respectively. A _control t=0_ and A _control t=2h_ are the absorbance values for the control at the beginning of the experiments and after 2 h assay, respectively.

#### 4.7.4. Reducing Power Activity

The reductive potential of ALE was determined following the method of Bounatirou et al. [45]. Different concentrations of ALE were mixed with phosphate buffer (2.5 mL, 0.2 M, and pH 6.6) and potassium ferricyanide (2.5 mL, 1% *w*/*v*). The mixture was incubated at 50 °C for 20 min. Trichloroacetic acid (10%, 2.5 mL) was added to the mixture and centrifuged for 10 min at 1200× *g*. The upper layer of the solution (2.5 mL) was mixed with distilled water (2.5 mL) and FeCl_3_ (0.5 mL, 0.1%), and the absorbance was measured using a spectrophotometer (700 nm). The extract concentration providing 0.5 of absorbance (IC_50_) was calculated by plotting the absorbance at 700 nm against the corresponding ALE concentration. BHT and ascorbic acid were used as reference compounds.

### 4.8. Polyphenols Quantification in ALE

#### 4.8.1. Total Polyphenol Content (TPC)

Total polyphenol content (TPC) of ALE was determined using Folin-Ciocalteu reagent according to the method of Catalano et al., with some modifications [46]. Briefly, ALE (0.1 mL) was dissolved in distilled water (3.9 mL). Folin–Ciocalteu reagent (0.1 mL) was added to the solution and allowed to stand for 3 min. After the addition of 1 mL of a 20% anhydrous sodium carbonate solution (*w*/*v*), the mixture was shaken actively then incubated for 1 h in the dark. The absorbance was measured at 765 nm. Total polyphenol content was determined using a standard curve prepared with tyrosol. The results are expressed as mg of tyrosol equivalents (TYE) per gram of dry matter (DM).

#### 4.8.2. Total Flavonoids Content

Total flavonoids content of ALE was determined according to the method described by Kim et al., with some modification [47]. Briefly, 200 µL of extract was mixed with 800 µL of distilled water in a test tube, and 60 µL of a 5% sodium nitrite solution was added. After 5 min, 40 µL of a 10% aluminum chloride solution was added and the mixture was allowed to stand for a further 5 min before 0.4 mL of Na_2_CO_3_ (1 M) was added. The mixture was brought to 0.5 mL with distilled water and mixed well. The absorbance was measured immediately at 510 nm using a spectrophotometer. Total flavonoid content was calculated from a calibration curve using catechin as a standard, and expressed as mg catechin equivalents (CTE) per g of DM.

#### 4.8.3. Flavanols Content

Flavanols content of ALE was determined by *p*-(dimethylamino)-cinnamaldehyde (DMACA) reagent using the optimized protocol established by Nigel and Glories [48]. The extract (0.2 mL) was mixed with 0.5 mL HCl (0.24 N in methanol) and 0.5 mL DMACA solution (0.2% in methanol). The mixture was allowed to react for 5 min at room temperature, and the absorbance was measured at 640 nm. The total flavanols content was calculated from a calibration curve, using catechin as a standard. The results are expressed as mg of catechin equivalents (CTE) per g of DM.

#### 4.8.4. Proanthocyanidins Content

Proanthocyanidins concentration was determined according to the method described by Waterman and Mole [49]. Butanol reagent was prepared by mixing 70 mg ferrous sulphate (FeSO_4_) with 5 mL concentrated HCl and made to 100 mL with *n*-butanol. The extract (0.1 mL) was mixed with 1.4 mL of butanol reagent. The mixture was heated at 95 °C in a water bath for 45 min. The mixture was then cooled, 0.5 mL *n*-butanol was added and the absorbance was measured at 550 nm. Results were expressed as cyanidin equivalents (CYE) per g of DM.

### 4.9. Phenols Characterization

Phenols composition of ALE was determined using UPLC-ESI-HRMS. Samples were run on an UPLC Dionex Ultimate 3000 (Thermo Fisher Scientific, Lissieu, France) equipped with a column Acquity UPLC BEH C_18_ (1.7 μm, 2.1 × 150 mm) (Waters, Guyancourt, France). The elution was performed using a binary gradient system consisting of acidified water (H_2_O/HCOOH, 99:1, *v*/*v*) and acetonitrile at a constant flow rate of 0.3 mL/min. The elution started with 5% acetonitrile and increased to 100% in 12 min. The injection volume of the sample was 10 µL. The detection wavelength was set at 280 and 320 nm with a diode-array UV detector. Mass spectrometry measurements were performed on a Waters Xevo G2Qtof (Waters, France). High resolution mass spectra were recorded in negative ion mode, under the following operating conditions: capillary voltage, 2.5 kV; cone voltage, 30 V; extraction cone, 2 V; ion source temperature, 130 °C. The spectra were acquired in the *m/z* range of 100–1200. Analyses were monitored using the Chromeleon software 6.60 (Dionex, COUNTRY).

### 4.10. Statistical Analysis

The results are presented as the mean ± standard deviation (SD) of three independent experiments. Statistical significance of differences between the groups was assessed by one-way analysis of variance (ANOVA) using Tukey’s test. The differences are considered significant when *p* value is ≤0.05. All statistical analyses were carried out using COSTAT software version 6.3.

## 5. Conclusions

The significant melanogenesis stimulation activity of ALE is correlated with its strong antioxidant capacity and may be related to its high content of polyphenols; a relationship that has been demonstrated by other studies for plants extracts. Hence, argan leaves may be a potential option for the treatment of hypopigmentation disorders and may be used as a bioactive component of self-tanning cosmetic products. Further studies would be needed to clearly define the molecular mechanism of ALE and to examine whether, topically applied, argan leaves can enhance human skin pigmentation under clinical or physiological conditions. We report in this study, for the first time, that in addition to previously reported phenolic compounds, gallic acid, (+)-gallocatechin, (−)-epigallocatechin, quercetin-3-*O*-glucuronide, quercetin-7-*O*-rhamnoside, and quercetin are also present in ALE.

## Figures and Tables

**Table 1:** Total phenol content and antioxidant activities of argan leaves extract (ALE) *.

	Parameters	ALE	BHT	Ascorbic Acid
Phenol content	TPC (TYE mg/g)	198.61 ± 0.20	-	-
	Flavonoids (CAE mg/g)	128.41 ± 1.58	-	-
	Flavanols (CAE mg/g)	59.59 ± 1.55	-	-
	Proanthocyanidins (CYE mg/g)	6.85 ± 0.13	-	-
Antioxidant activities (IC_50_ mg/mL)	DPPH	0.508 ± 0.016 ^c^	0.147 ± 0.001 ^b^	0.071 ± 0.003 ^a^
	Reducing power	0.316 ± 0.011 ^b^	0.354 ± 0.011 ^c^	0.070 ± 0.011 ^a^
	β-carotene	1.679 ± 0.070 ^b^	0.050 ± 0.001 ^a^	^-^
	ABTS	0.373 ± 0.001 ^c^	0.340 ± 0.001 ^b^	0.078 ± 0.001 ^a^

Legend: ALE: argan leaves extract; TPC: Total phenol content; TYE: Tyrosol equivalent; CAE: Catechin equivalent; CYE: Cyanidin equivalent; BHT: Butylated hydroxytoluene. * Values represent the mean ± standard deviation of three independent experiment; values in a row with different letter are significantly different (*p* ≤ 0.05) (ANOVA Tukey’s test).

**Table 2:** Characterization of argan leaves phenolic compounds by UPLC-ESI-HRMS.

Peak Number	Retention Time, min (Area%)	Compound Identity	Molecular Formula	UPLC UV/Vis λmax (nm)	Experimental *m*/*z* (M − H)^−^	Theoretical *m*/*z* (M − H)^−^	References
1	0.992 (1.36)	Gallic acid	C_7_H_6_O_5_	-	169.014	169.0135	[12]
2	1.204 (2.19)	(+)-Gallocatechin	C_15_H_14_O_7_	-	305.0654	305.0657	[12,13]
3	1.49 (3.10)	(−)-Epigallocatechin	C_15_H_14_O_7_	-	305.0657	305.0657	[12,13]
4	1.736 (3.18)	(+)-Catechin	C_15_H_14_O_6_	-	289.0707	289.0708	[14]
5	1.801 (2.95)	(−)-Epicatechin	C_15_H_14_O_6_	-	289.0707	289.0708	[14]
6	1.86 (8.37)	Quercetin-3-*O*-Glucuronide	C_21_H_18_O_13_	231.9–253.3–347.1	477.1035	477.0663	[15]
7	2.03 (35.67)	Myricetin-3-*O*-Galactoside	C_21_H_20_O_13_	250.9–266.2–326.6	479.0825	479.0819	[16,17]
8	2.201 (4.35)	Unknown	-	-	479.082	-	-
9	2.266 (19.39)	Myricitrin	C_21_H_20_O_14_	-	463.0877	463.087	[16,17]
10	2.408 (1.23)	Quercitrin	C_21_H_20_O_11_	250.3–266.2–326.6	447.093	447.0921	[16,17]
11	2.538 (3.96)	Quercetin-7-*O*-rhamnoside	C_21_H_20_O_11_	-	447.0929	447.0921	[18]
12	2.663 (0.33)	Myricetin	C_15_H_10_O_8_	222.2–304.3	317.029	317.0294	[17]
13	2.774 (2.30)	Rutin (Quercetin-3-*O*-rutinoside)	C_30_H_26_O_14_	257.0–313.0	609.1234	609.1236	[15]
14	3.073 (0.95)	Quercetin	C_15_H_10_O_7_	254.5–370.1	301.0345	301.0345	[19]

## Sample Availability

Samples of ALE are available from BT.

## Abbreviations

ALE	argan leaves extract
ABTS	2,2’-azinobis-3-ethylbenzothiazoline- 6-sulphonate
BHT	butylated hydroxytoluene
CTE	catechin equivalents
CYE	cyanidin equivalents
DCT	dopachrome tautomerase
DPPH	1,1-diphenyl-2-picrylhydrazyl radical
l-DOPA	l-3,4-dihydroxyphenylalanine
DM	dry matter
GAE	gallic acid equivalents
MITF	microphthalmia-associated transcription factor
α-MSH	alpha-melanocyte stimulating hormone
MTT	3-(4,5-dimethylthiazol-2-yl)-2,5-diphenyl-tetrazolium bromide
PVDF	polyvinylidene difluoride membrane
QE	quercetin equivalents
RIPA	radioimmunoprecipitation
SDS-PAGE	sodium dodecyl sulfate-polyacrylamide gel electrophoresis
TPC	total phenols content
TFC	total flavonoids content
TRP1	tyrosinase-related protein 1
TYE	tyrosol equivalents
TYR	tyrosinase
UPLC-ESI-HRMS	ultra-performance liquid chromatography–electrospray-ionization-high resolution mass spectrometry
